# Development and Performance Study of Continuous Oil–Water Separation Device Based on Superhydrophobic/Oleophilic Mesh

**DOI:** 10.3390/nano15060450

**Published:** 2025-03-16

**Authors:** Tianxin Chen, Yue Wang, Jing Li, Liang Zhao, Xingyang Zhang, Jian He

**Affiliations:** 1 Research Institute of Safety, Environmental Protection and Technical Supervision of Petro China Southwest Oil and Gas Field Company, Chengdu 610095, China; chentianxin@petrochina.com.cn (T.C.);; 2 School of Chemical Engineering, Sichuan University, Chengdu 610065, China

**Keywords:** spray method, superhydrophobic, oleophilic, oil–water separation, residence time

## Abstract

Oil–water separation is an important method for treating oily wastewater and recovering oil resources. Based on the different affinities of superhydrophobic surfaces to water and oil, long-term oil–water separation devices with low-energy and high efficiency can be developed through the optimization of structure and process parameters. Superhydrophobic coatings were prepared on stainless-steel mesh surfaces using a spray method to construct single-channel oil–water separation equipment with superhydrophobic/oleophilic meshes, and the effects of structural and process parameters on separation efficiency were systematically investigated. Additionally, a multi-channel oil–water separation device was designed and fabricated to evaluate the feasibility and stability of long-term continuous operations. The optimized single V-shaped channel should be horizontally placed and made from 150-mesh stainless-steel mesh folded at an angle of 38.9°. For the oil–water mixtures containing 20 wt.% oil, the oil–water separation efficiencies for single and two-stage separation were 92.79% and 98.96%, respectively. After 36 h of continuous operation, the multi-channel separation device achieved single-stage and two-stage separation efficiencies of 94.60% and 98.76%, respectively. The maximum processing capacity of the multi-channel device reached 168 L/h. The modified stainless mesh can remain stable with a contact angle (CA) higher than 150° to water for 34 days. The average residence time and contact area during the oil–water separation process significantly affect separation efficiency. By optimizing oil–water separation structures and process parameters, and using a superhydrophobic spray modification method, separation efficiency can be improved while avoiding the generation of secondary pollutants.

## 1. Introduction

Oily wastewater is widely distributed in the field of oil and gas extraction, which can cause environmental damage and resource waste without further treatment [1]. At present, the existing treatment methods for oily wastewater vary depending on the environmental conditions and oil content; they include gravity separation, flocculation separation, membrane separation, and adsorption separation [2,3,4,5,6]. These technologies have demonstrated excellent oil–water separation performance in practical production, but they also face issues such as low treatment efficiency and high operating costs [7]. As solid waste is generated during the flocculation process, it further increases the complexity of the process [6]. In particular, some traditional oil–water separation materials exhibit the characteristic of simultaneously adsorbing both oil and water, which not only reduces the separation efficiency but also makes post-adsorption material disposal difficult. Based on advanced oleophilic and hydrophobic materials as oil–water separation media, developing a new oil–water separation technologies can effectively improve the separation efficiency of oily wastewater, and enhance oil and gas resource recovery efficiency [8,9]. Due to the superhydrophobicity and superoleophilicity in the air, superhydrophobic materials can be processed into two-dimensional mesh or three-dimensional porous sponge for oil–water separation, making these a research hotspot in oil–water separation technology.

The development of superhydrophobic materials is inspired by biomimetic science, primarily referring to surfaces with a contact angle (CA) greater than 150° and a sliding angle less than 10° [10,11]. According to their study of the unique wettability of lotus leaves, water striders, spiders, and other creatures, Jiang et al. [12,13] identified that ultra-low surface energy and micro–nanostructures are the key factors for constructing superhydrophobic surfaces. After that, various superhydrophobic surfaces were prepared using templates, chemical corrosion, anodization, thermal oxidation, and spraying methods, which effectively drove the advancement of interfacial science. Moreover, based on the special properties of superhydrophobic surfaces, superhydrophobic materials have been applied in numerous fields, including self-cleaning, anti-icing, oil–water separation, anti-corrosion, and granulation [14,15,16,17,18]. In turn, this also promoted the development of industrial production. In 2004, Jiang’s group developed a superhydrophobic/superoleophilic mesh using a simple spraying method, which exhibited excellent oil–water separation performance [19]. Researchers have since used various methods to fabricate two-dimensional (2D) superhydrophobic meshes and three-dimensional (3D) superhydrophobic porous adsorption materials, achieving efficient oil recovery while minimizing the influence of water [20,21].

Even though superhydrophobic/superoleophilic materials have shown great potential in applications for oil–water separation, they also remain largely confined to laboratory research and are difficult to use in industrial production. The main reason is that the wettability of superhydrophobic/superoleophilic materials primarily relies on their unique surface structures, which suffer from poor mechanical stability in practical applications. Additionally, the modified components used in the construction of superhydrophobic coatings are prone to dissolution by oil, resulting in coating damage and high economic costs [10,22,23]. Moreover, in actual production or daily life, the density of oil is usually lower than that of water, resulting in oil floating on the water. When superhydrophobic meshes are used for oil–water separation, the bottom of the mesh can be easily blocked by water, preventing the oil from coming into contact with the mesh and reducing the efficiency of oil–water separation. Furthermore, as the pressure of the water increases, it also can penetrate the superhydrophobic mesh [24,25]. Therefore, the development of continuous oil–water separation devices based on superhydrophobic materials faces challenges. The effective way to solve the problem of oil-water entrainment is to design a reasonable oil–water separation channel, which can increase the contact between the mesh and the oil–water mixture for separation.

In this study, a stable superhydrophobic surface was developed using stainless-steel mesh as the substrate, combined with polytetrafluoroethylene (PTFE)/polyvinylidene fluoride (PVDF) micro–nano powder and epoxy adhesive. Stainless-steel wire mesh is characterized by its corrosion-resistance and low cost, which can effectively decrease the cost of the oil–water separation process. A simple and scalable spray-coating method was employed as the modification technique. The stainless-steel mesh was shaped into V-shaped grooves, and the effects of the geometry and process parameters of the single channel on the oil–water separation efficiency were systematically explored. The optimized parameters were determined through a carefully evaluation. Furthermore, a scaled-up separation device was designed and fabricated based on the optimized parameters. The performance of the device was assessed by performing a long-term, continuous operation in separating oil–water mixtures. The stability of the surface resistant to the oil was also evaluated.

## 2. Materials and Method

### 2.1. Materials and Characterization

The epoxy adhesive was purchased from Hunan Brothers New Materials Co., Ltd. (Changsha, China). The micro/nano polytetrafluoroethylene (PTFE) powder and nano-scale polyvinylidene fluoride (PVDF) powder were obtained from Dongguan Huachuangxin Plastics Co., Ltd. (Dongguan, China). The perfluorosilane was obtained from Shanghai Titan Scientific Co., Ltd. (Shanghai, China). The kerosene was of analytical grade and obtained from Shanghai Aladdin Biochemical Technology Co., Ltd. (Shanghai, China). The ethyl acetate was purchased from Shanghai Titan Scientific Co., Ltd. The stainless-steel mesh of 100, 120, and 150 mesh were purchased from Runda Mesh Industry in Hengshui, Hebei Province.

The morphology and composition of the samples were analyzed via scanning electron microscopy (SEM) and energy-dispersive X-ray spectrometry (EDS, JSM-7610F, JEOL, Tokyo, Japan). A contact angle goniometer (Shanghai Zhongchen Digital Technology Equipment Co., Ltd., Shanghai, China, JC2000C1) was used to evaluate the wettability of the surface. The chemical composition of the surface was measured using Fourier transform infrared spectroscopy (FTIR) (Spectrum Two Li10014, PerkinElmer, Waltham, MA, USA) and X-ray photoelectron spectrometer (XPS) (Escalab250Xi, Thermo Scientific, Waltham, MA, USA) with an Al Kα (E = 1486.6 eV).

### 2.2. Preparation of Superhydrophobic Surface

The composition of the paint used to prepare the superhydrophobic surface included micro/nano-scale powder, adhesives, and dispersants. The micro/nano-scale powder was composed of 1 g of micron-scale Polytetrafluoroethylene (PTFE) powder, 1.5 g of nano-scale PTFE powder, and 0.5 g of PVDF powder. The adhesives were prepared with 1 g of E44 epoxy resin, and 0.2 g of curing agent. The addition of 0.45 g of perfluorosilane (CAS No.: 83048-65-1) was used to enhance the hydrophobic ability. Then, the above materials were dispersed into 30 g of ethyl acetate. After ultrasonic treatment for 30 min, the superhydrophobic stainless-steel mesh was fabricated using a spray-drying process, with a spraying distance of 15–20 cm. The V-shaped stainless-steel meshes with 100, 120, and 150 mesh sizes were cleaned for 30 min to remove surface contaminants. The prepared coating mixture was then uniformly sprayed onto the surface of the stainless-steel mesh using a spray gun. The paint was applied to the stainless-steel mesh at a density of 0.1–0.2 mL/cm^2^. Finally, the sprayed meshes were placed in an oven at 80 °C and cured for 3 h. The CAs of water and kerosene on the prepared superhydrophobic surface were measured to evaluate the wettability of the coatings.

### 2.3. Oil–Water Separation Process

**Figure 1:**
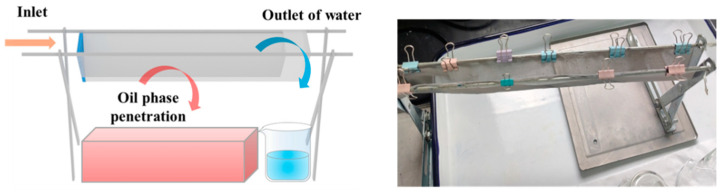
Structure and physical diagram of oil–water separation device with single channel V-shaped network.

In the oil-water separation process, an oil–water mixture with a total mass of 500 g was prepared, where the mass fraction of oil in the mixture ranged from 10 wt% to 30 wt%. The mixture was stirred at a speed of 600–1000 rpm for 5 min using a magnetic stirrer. To maintain the stability of the oil–water mixture, the magnetic stirring was in continuous operation during the oil–water separation process. Then, the prepared oil–water mixture was transferred to the oil–water separation tank using a peristaltic pump. The rotation speed of the peristaltic pump was controlled at 30–100 rpm, with the flow rate calibrated to 21.1 L/h at 100 rpm. The experimental setup for a single-channel device is shown in Figure 1. The opening and tilt angle of the channel could be adjusted using a support frame to control the residence time and contact area of the mixture within the channel. The separated oil and water at the outlet were collected, weighed, and recorded. The separation efficiency (*η*) was calculated using the following equation:

(1) $$\eta =\frac{m-m_{1}}{m_{0}}\;\times \;100\%$$

where *m* is the total mass of the oil–water mixture before separation (g), *m*_0_ is the mass of the kerosene before separation (g), and *m*_1_ is the mass of the separated mixture at the outlet (g). The single-channel oil–water separation device consisted of a support, a 15 cm long V-shaped channel, and a polymethyl methacrylate (PMMA) baffle (thickness: 0.8 cm) at the inlet of channel to prevent backflow of the oil–water mixture. The width of the V-shaped channel could be adjusted by modifying the angle of the support, allowing for the evaluation of separation efficiency in channels with different structures. During the separation process, the oil–water mixture was transported to the channel inlet using a peristaltic pump. The oil penetrated the stainless-steel mesh and drips into the oil collection tank, while water flows out from the end of the channel. Prior to the separation process, the device was pre-wetted with pure water and pure oil to reduce errors caused by residual liquid in the device. The separation time was controlled throughout the experiment. Based on the optimized oil–water separation parameters of a single channel, in order to further improve the processing capacity of the equipment for oil–water mixtures, this study also designed and fabricated a multi-channel continuous oil–water separation device. Additionally, a preliminary investigation was carried out on the efficiency of the scaled-up oil–water separation.

## 3. Result and Discussion

### 3.1. Characterization of Superhydrophobic Mesh

**Figure 2:**
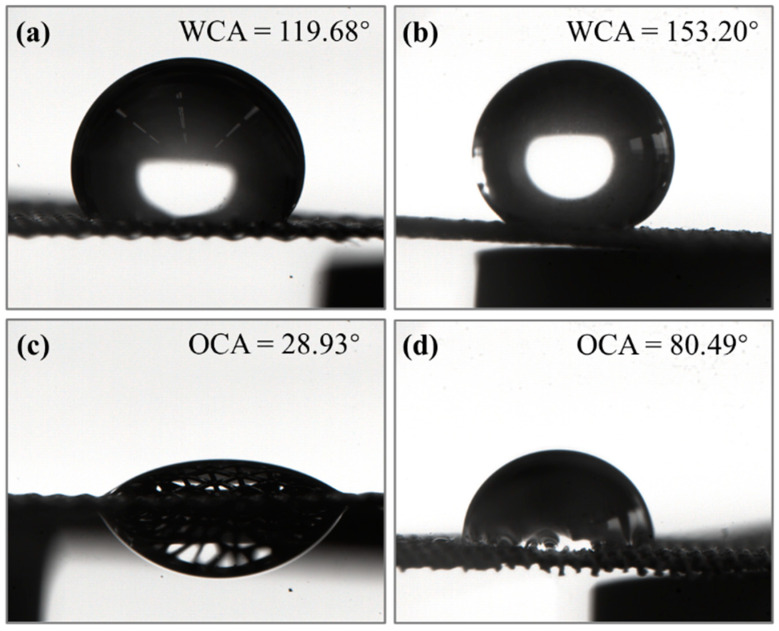
Test results of contact angle: WCAs of (**a**) unmodified and (**b**) modified stainless-steel mesh, OCAs of (**c**) unmodified and (**d**) modified stainless-steel mesh.

To investigate the wettability of the surface, the CAs of water and oil on the stainless- steel mesh before and after modification was measured, as shown in Figure 2. The results showed that the CA of water on the unmodified stainless-steel mesh was 119.68°, indicating hydrophobicity to water. This may be attributed to the presence of oil residue on the surface during the manufacturing process, which caused the surface to exhibit hydrophobic properties. After further spray modification, the CA of water increased to 153.2°, demonstrating an excellent superhydrophobicity. For kerosene, the unmodified stainless-steel mesh exhibited a CA of 28.93°, indicating oleophilicity, allowing kerosene to penetrate the metal mesh. After the modification, the CA of kerosene on the modified mesh increased to 80.49°. The oleophilicity of the stainless steel mesh was reduced. The modified stainless-steel mesh exhibited a significant difference in CA for water and oil. This superhydrophobic and oleophilic property is crucial for achieving oil–water separation.

**Figure 3:**
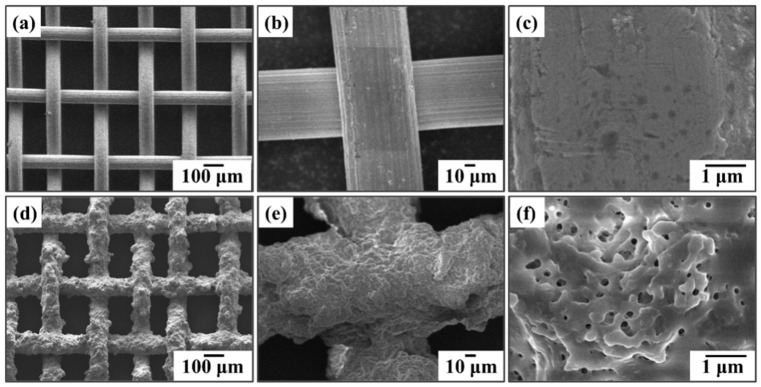
SEM images of unmodified and modified samples: pristine mesh of (**a**) ×100, (**b**) ×500, (**c**) ×20,000; superhydrophobic mesh of (**d**) ×100, (**e**) ×500, (**f**) ×20,000.

**Figure 4:**
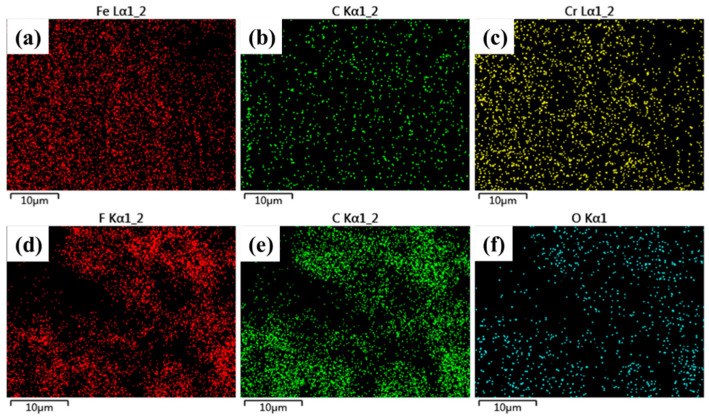
EDS mappings of unmodified and modified samples: pristine mesh of (**a**) Fe, (**b**) C, (**c**) Cr; superhydrophobic mesh of (**d**) F, (**e**) C, (**f**) O.

The microstructure and composition of the mesh before and after modification were analyzed using SEM and X-ray energy dispersive spectroscopy (EDS). The results are shown in Figure 3 and Figure 4. From Figure 3a and Figure 4a, it can be seen that the surface of the unmodified mesh is smooth and flat. The elements of the surface consist only of iron, chromium, and carbon, which originate from the stainless-steel mesh. Even at a magnification of 20,000×, only tiny protrusions and grooves can be observed on the surface. In contrast, after the spray coating modification, Figure 3b shows that the surface morphology of the stainless-steel mesh changes significantly. The metal wires of the stainless-steel mesh are uniformly coated with the modified layer, and a rough structure composed of micro/nano-particles was formed on the stainless-steel mesh. At high magnification, nanopores can also be observed on the modified surface; these are formed during the curing process as a result of solvent evaporation. The EDS analysis in Figure 4b shows the presence of fluorine and oxygen on the surface of the modified stainless-steel mesh. The fluorine originates from the micro/nano-scale PTFE particles, PVDF powder, and perfluorosilane, while the oxygen originates from the epoxy resin. These results indicate that the spray-coating and curing process successfully fixed the micro/nano-scale hydrophobic particles onto the mesh wires, forming a modified coating that transformed the wettability of the mesh into a superhydrophobic/oleophilic characteristic.

**Figure 5:**
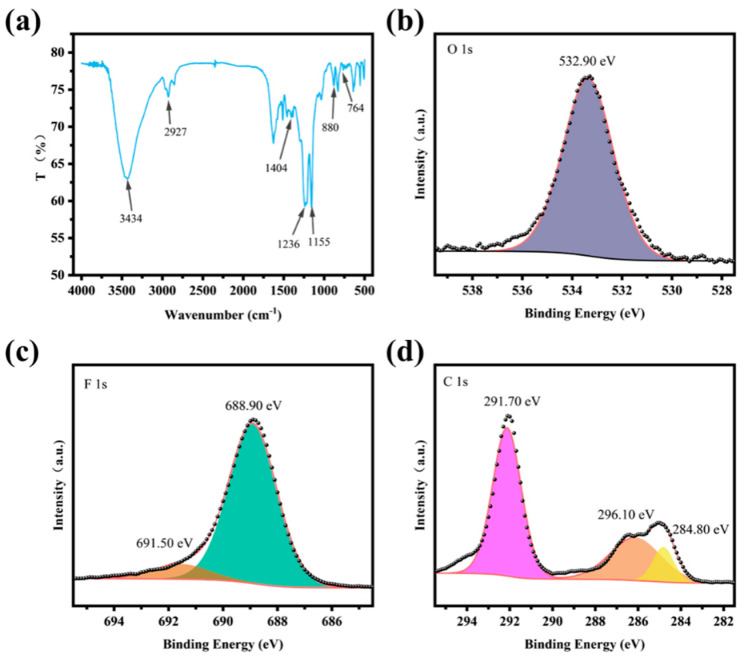
FTIR and XPS test results of the coating: (**a**) the FTIR spectrum, (**b**) the 1s spectrum of O, (**c**) the 1s spectrum of F, and (**d**) the 1s spectrum of C.

To further confirm the chemical composition of the surface, an FT-IR analysis was conducted on the modified surface, as shown in Figure 5a. The result indicates that the absorption peak at 3434 cm^−1^ corresponds to hydroxyl groups, and the peak at 2927 cm^−1^ belong to methylene groups. Moreover, the peak at 1404 cm^−1^ should belong to C-F bonds, and the peaks at 1236 cm^−1^ should be ascribed to the stretching vibration of C-O bonds. The peaks at 878 cm^−1^ and 764 cm^−1^ correspond to the β-phase and α-phase of PVDF, respectively [26]. Additionally, the X-ray photoelectron spectroscopy (XPS) analysis was performed on the modified mesh. The spectra of different characteristic elements are shown in Figure 5b–d. A peak corresponding to O1s was observed at a binding energy of 532.90 eV, originating from the C-O-C bonds in the epoxy resin. Peaks corresponding to fluorine (F1s) were observed at binding energies of 688.90 eV and 691.50 eV, which should belong to the C-F bonds in PTFE and PVDF. Peaks at C1s were observed at binding energies of 284.80 eV, 286.10 eV, and 291.70 eV, which originate from the C-C bonds in PTFE and PVDF. The analysis of the functional groups confirmed that the coating is primarily composed of epoxy resin, PVDF, and PTFE, which were successfully fixed to the surface of the stainless-steel mesh.

### 3.2. Effect of Structural Parameters on Separation Process

**Figure 6:**
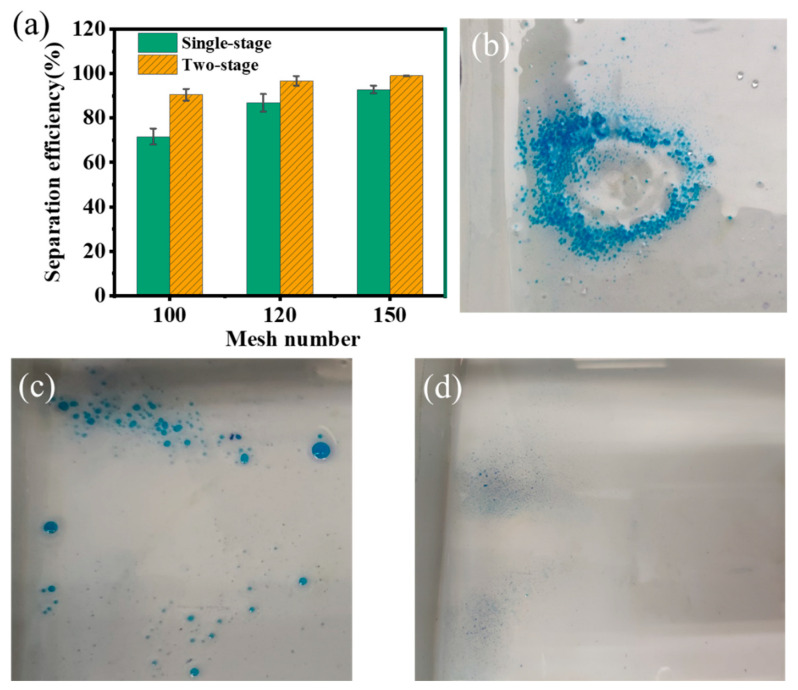
The separation efficiency of superhydrophobic mesh with different mesh numbers: (**a**) the separation efficiency of meshes, the appearance of oil using superhydrophobic mesh with mesh numbers of (**b**) 100, (**c**) 120 and (**d**) 150.

First of all, the oil–water separation performance of a single channel was first investigated to optimize the structural and process parameters. The stainless-steel meshes with a pore size at 100-mesh, 120-mesh, and 150-mesh were folded into a V-shape. Then the mesh was modified with hydrophobic paints. After that, the modified meshes were dried and fixed in the separation device to study the effect of pore size on the separation efficiency. The results are shown in Figure 6a–d. As the pore size decreased, the separation efficiency increased from 71.61% (100-mesh) to 92.79% (150-mesh) for the first time. After two separation processes, the efficiency increased from 90.41% to 98.96%. Additionally, a small amount of water passed through the pore of the mesh into the separated oil using the separating meshes with a pore size of 100-mesh and 120-mesh. However, when the oil–water mixture was separated using the meshes with a pore size of 150-mesh, no water can be observed in the separated oil. The reason for this phenomenon is that the pressure of the oil–water mixture entering the channel was higher than the interception ability of the mesh pores, allowing some water to permeate through the pores. However, as the pore size decreased, the interception ability of the modified mesh was enhanced. Despite the increased inlet pressure, the mesh maintained excellent interception capability. Nevertheless, if the pore size of the mesh is too small, the oil may fail to pass through the mesh in a timely manner, leading to oil being carried away by the water and reducing the separation efficiency.

**Figure 7:**
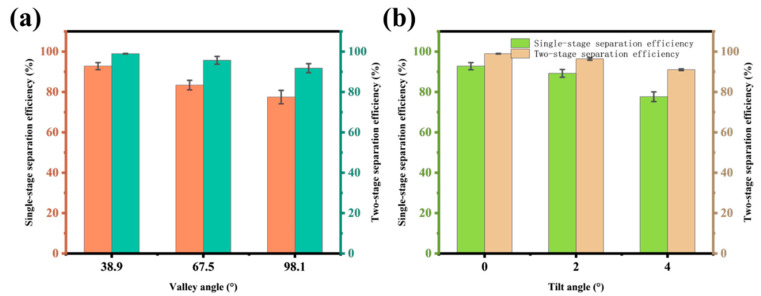
The influence of valley angle and tilt angle on separation efficiency: the separation efficiency of different (**a**) valley and (**b**) tilt angles.

Prior studies have shown that the efficiency of oil–water separation is related to both the residence time and the contact area [23,24]. The intersection angle of the V-shaped separation mesh not only affects the height of the oil–water mixture in the channel but also influences the spreading area of oil on the liquid. Therefore, by using a triangular support frame to adjust the angle of channel, the separation efficiency under three different angles was investigated (Figure 7a). The experimental results showed that as the angle increased from 38.9° to 98.1°, the primary separation efficiency decreased from 92.79% to 77.47%, and the secondary separation efficiency decreased from 98.96% to 91.77%. This indicates that reducing the trough angle can improve separation efficiency. However, the space of the channel becomes restricted as the angle decreases. To ensure both high channel volume and separation efficiency, a fixed angle of 38.9° was selected for subsequent research.

By adjusting the tilt angle of the channel, the flow rate of the oil–water mixture can be controlled, which can be used to regulate the residence time of the oil–water mixture. As shown in Figure 7b, the results indicate that as the tilt angle increased, the separation efficiency gradually decreased. The single-stage separation efficiency decreased from 92.79% (horizontal placement) to 77.65% (tilt angle of 4°), while the two-stage separation efficiency decreased from 98.96% to 91.03%. Therefore, the larger the tilt angle, the poorer the separation performance and the lower the separation efficiency. This is because increasing the tilt angle of the channel causes the flow rate of the oil–water mixture to increase under the influence of gravity, thereby reducing the residence time. As a result, some of the oil enters the water collection tank before it has been fully separated, leading to a decrease in separation efficiency.

### 3.3. Effect of Operating Parameters on Separation Process

**Figure 8:**
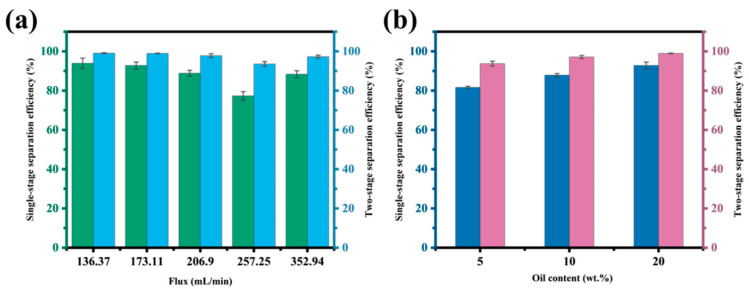
The separation efficiency of different (**a**) flux and (**b**) oil content.

The process parameters are additional important factors affecting separation efficiency. Therefore, separation efficiency was investigated by adjusting the inlet flow rate, and the results are shown in Figure 8a. It can be seen that with the increase in flow rate, the separation efficiency of both one-stage and two-stage processes was decreased at first and then increased. When the inlet flow rate was 257.25 mL/min, the separation efficiency was at its lowest point, with single-stage separation efficiency at 77.31% and two-stage separation efficiency at 93.58%. This is because when the inlet flow velocity increases, the turbulence of the oil–water mixture in the channel and the disturbance of the liquid surface are enhanced. In turn, the contact probability between the oil and the wall of the channel was also increased, which improved the separation efficiency.

Moreover, the performance of the superhydrophobic-stainless-steel mesh was evaluated for the oil–water mixtures with different concentrations of oil. The oil content in the oil–water mixtures was fixed at 5 wt.%, 10 wt.%, and 20 wt.%. The results in Figure 8b showed that the separation performance increased with the increase in oil content. The separation efficiency of single-stage process rose from 81.65% (5 wt.%) to 92.79% (10 wt.%), while the separation efficiency of the two-stage process increased from 93.75% to 98.96%. The impact of oil content on separation performance is mainly due to the difference in the thickness of the oil layer within the channel under varying oil concentrations. The higher the oil concentration, the thicker the oil layer in the separation channel. As a result, the static pressure differences and contact areas are also enhanced, which lead to increased oil permeability and improved separation efficiency.

### 3.4. Designed and Prepared Multi-Channel Equipment

**Figure 9:**
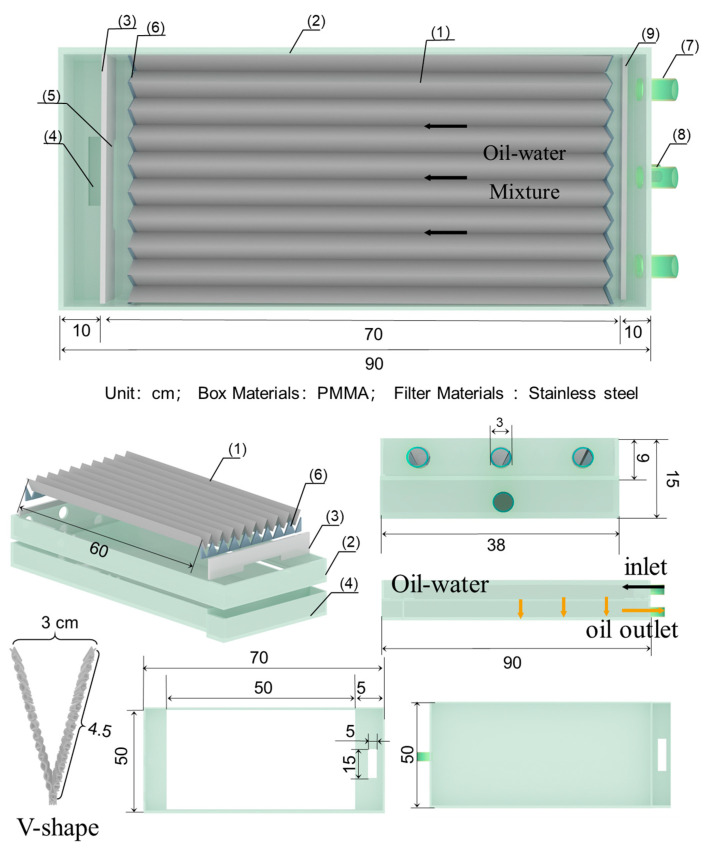
Design details of parallel multi-channel oil–water separation device based on superhydrophobic/oleophilic separation mesh: 1. Oil–water separation mesh; 2. Oil–water separation tank; 3. Baffle; 4. Inlet of the secondary separation unit; 5. Outlet; 6. Triangular baffle; 7. Inlet pipe; 8. Oil outlet pipe; 9. Baffle.

To increase the processing capacity of the equipment for oil–water mixtures, a multi-channel device was designed and manufactured based on parameters from the single-channel device for continuous oil–water separation, as shown in Figure 9. The device consisted of eight V-shaped channels connected in parallel, with a trough angle of 39°. The length of the channel was fixed at 60 cm. Two peristaltic pumps were used to transport the oil–water mixture to the inlet of the channels. The oil–water mixture overflowed into each channel, with the oil in the mixture permeating through the mesh into the oil tank and was collected from the left outlet. Meanwhile, the water flowed along the channel to the end and was collected from the right outlet. The maximum processing capacity of the single-channel device can reach 21 L/h. To investigate the reduction of separation efficiency caused by scaling up, the maximum processing capacity was 168 L/h. The main structure included an oil–water separation tank, V-shaped separation meshes, baffles, overflow weirs, an oil collection tank, and a water collection tank.

**Figure 10:**
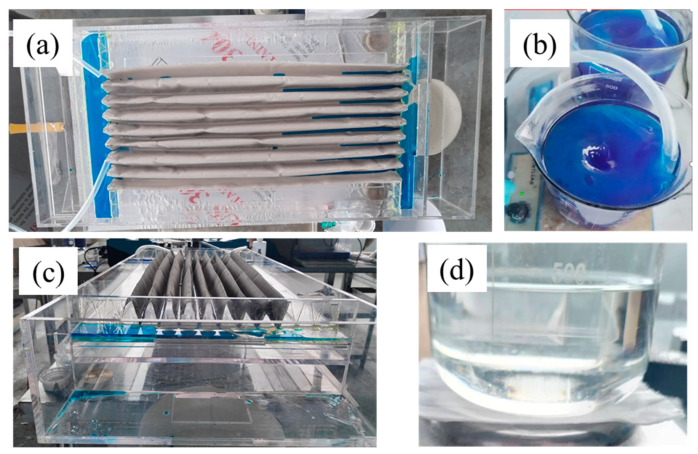
Appearance of (**a**) multi-channel separation device based on V-shaped mesh, (**b**) oil–water mixture, (**c**) side view and (**d**) separated oil.

The appearance of the equipment is shown in Figure 10a,c. A mixture of kerosene and water was used for the separation, with the water dyed using methylene blue to make it clearly observed in the oil–water separation process. The kerosene/water mixture was vigorously stirred during transport; the morphology of the wastewater under intense stirring conditions is shown in Figure 10b. The mixture was transported using two peristaltic pumps operating at a maximum speed of 100 rpm/min. The mixture overflowed into the channels from the overflow tank. The pre-wetting process for the channels before separation significantly improved the efficiency of the initial stage. Kerosene permeated through the separation mesh and flowed out from the back of the channel, while the dyed water exited from the sides. As shown in Figure 10d, the separated oil was observed to be clear and transparent, with no residual water.

**Figure 11:**
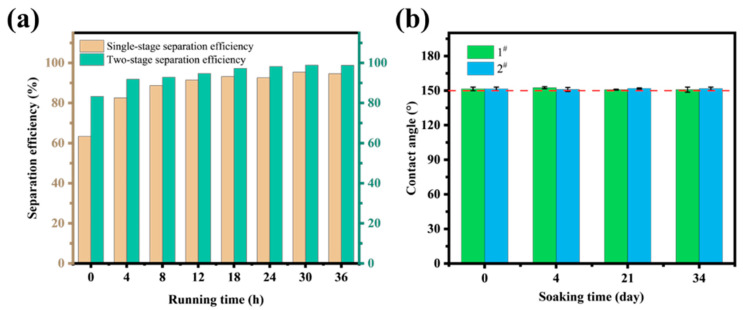
Continuous long-term performance and stability evaluation of multi-channel separation device: (**a**) separation efficiency, (**b**) contact angles after soaking for different number of days.

The stability of the equipment was evaluated through long-term continuous separation experiments. The separation efficiency of the equipment was analyzed, and the results are shown in Figure 11a. The single-stage and two-stage separation efficiencies gradually increased from 67.37% and 83.30% to 94.60% and 98.76% after 36 h of continuous operation. The gradual improvement in separation performance is attributed to the enhanced wetting degree of the oil on the separation mesh as the separation process continues. This improved wetting allows the oil phase to quickly penetrate the mesh and separate efficiently upon contact with the wall of the channel, thereby gradually increasing the separation efficiency. To further investigate the stability of the superhydrophobic coating in oil, considering that the modified coating is composed of polymer materials, the modified stainless-steel mesh was soaked in two different condensate oils. The main components of these condensate oils were C5–C11 alkanes. The results indicate that the superhydrophobic surface can maintain its stability after 34 days of immersion, with contact angles remaining above 150° (Figure 11b). This stability ensures the reliability and durability of the oil–water separation channel for long-term continuous operation. Moreover, the experimental equipment designed in this study is easy to integrate and can be scaled up to increase oil–water separation capacity and efficiency, making it highly advantageous for developing compact oil–water separation systems.

### 3.5. Mechanism for Oil/Water Separation

**Figure 12:**
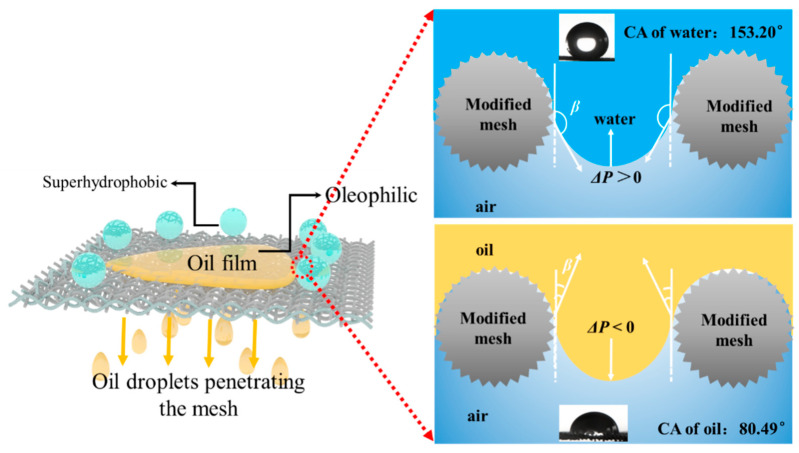
Schematic diagram of the wetting state and oil–water separation mechanism of oil and water in contact with stainless-steel mesh modified by spraying.

The mechanism for separating oil–water mixtures using the superhydrophobic/oleophilic mesh is illustrated in Figure 12. Due to the modified mesh exhibiting affinity for oil and strong repellence to water, the oil can permeate through the mesh pores while the water is retained, thus achieving the separation of the oil and water phases. The driving force for the oil to pass through the modified mesh is derived from the capillary pressure (Δ*p*) and the hydrostatic pressure (*p*) of the oil layer, which are calculated using Equations (2) and (3), respectively [27]:

(2) $$\Delta p=\frac{-l\gamma \cos\beta }{A}$$

(3) p=ρgh

where *l* is the perimeter of the capillary channel, in meters (m). The *γ* is the surface tension of the liquid, in newtons per meter (N/m). The *β* in Equation (2) is the contact angle of the liquid. *A* is the pore area of the mesh, in square meters (m^2^), and *ρ* is the density of the liquid, in kilograms per cubic meter (kg/m^3^). The *h* in Equation (3) is the thickness of the liquid layer (m). When the oil contacts the mesh pores, *β* is 80.49°, and cos*β* is greater than zero, resulting in a negative (Δ*p*). Therefore, the capillary pressure drives the oil to penetrate through the mesh pores. Conversely, when the water contacts the mesh pores, the contact angle of *β* is 153.20°, and cos*β* is less than zero, resulting in a positive (Δ*p*), where the capillary pressure blocks the water from passing through the pores of the mesh. This enables the selective separation of the oil and water mixture.

## 4. Conclusions

In this study, a V-shaped channel was prepared with a modified mesh, which was prepared by modifying the surface of a stainless-steel mesh using a spraying method. The paint was uniformly distributed on the wires of the mesh; the modified surface exhibited excellent superhydrophobicity, with a water contact angle of 153.20°. The structural parameters of a V-shaped single-channel used for oil–water separation were optimized. and the effect of process parameters and oil content on the separation efficiency were studied. When the mesh sizes were 100 and 120, a small amount of water penetrated through the pores of the mesh, which reduced the separation efficiency. Taking into account both separation efficiency and separation flux, the optimal mesh pore size was 150 mesh, achieving single-stage and two-stage oil separation efficiencies of 92.79% and 98.96%, respectively. The valley angle of the V-shaped mesh had a significant impact on separation efficiency. A smaller angle resulted in higher separation efficiency, but also reduced the flow rate. To keep the balance between the separation efficiency and flux, the optimal valley angle was determined to be 38.9°. With the increase of channel inclination, the residence time of oil–water mixture in the channel was reduced, which also decreased the separation efficiency. When the inlet flow rate was 352.94 mL/min, the single-stage and two-stage separation efficiencies were 88.36% and 97.31%, respectively. In addition, a multi-channel device was designed and manufactured, and its continuous performance for separating oil and water evaluated. The results showed that the equipment exhibited excellent operational stability. After 36 h of continuous separation, the single-stage and two-stage separation efficiencies for the oil phase were 94.60% and 98.76%, respectively. This study provides a foundation for the design and application of oil–water separation devices based on superhydrophobic materials, offering practical solutions for the application of superhydrophobic materials in industrial production.

## Data Availability

The original contributions presented in this study are included in the article. Further inquiries can be directed to the corresponding author.
